# Iron oxide nanoparticles suppress the production of IL-1beta via the secretory lysosomal pathway in murine microglial cells

**DOI:** 10.1186/1743-8977-10-46

**Published:** 2013-09-18

**Authors:** Hsin-Ying Wu, Min-Chun Chung, Chia-Chi Wang, Chung-Hsiung Huang, Hong-Jen Liang, Tong-Rong Jan

**Affiliations:** 1 Department and Graduate Institute of Veterinary Medicine, School of Veterinary Medicine, National Taiwan University, No.1, Sec. 4, Roosevelt Road, Taipei 10617, Taiwan; 2 School of Pharmacy, Kaohsiung Medical University, No. 100, Shih-Chuan 1st Rd, Kaohsiung 80708, Taiwan; 3 Department of Food Science, Yuanpei University, No.306, Yuanpei Street, Hsinchu 30015, Taiwan

**Keywords:** Cathepsin B, Interleukin-1β, Iron oxide nanoparticle, Lipopolysaccharide, Microglia, Secretory lysosome

## Abstract

**Background:**

Superparamagnetic iron oxide nanoparticles (IONPs) have been used as magnetic resonance imaging contrast agents for various research and diagnostic purposes, such as the detection of neuroinflammation and blood-brain-barrier integrity. As the central resident macrophage-like cells, microglia are responsible for managing foreign agents invading the CNS. The present study investigated the direct effect of IONPs on the production of pro-inflammatory cytokines by murine microglia stimulated with lipopolysaccharide (LPS).

**Methods:**

Primary murine microglial cells were pretreated with IONPs (1–50 μg Fe/mL) for 30 min and then stimulated with LPS (100 ng/mL) for 24 h. Confocal microscopy is used to visualize the intracellular IONP distribution and secretory lysosomes after staining with LysoTracker and Rab27a, respectively. The production of interleukin (IL)-1β and tumor necrosis factor (TNF)-α was quantified by ELISA. The activity of IL-1β converting enzyme (ICE) and TNF-α converting enzyme (TACE) was measured by fluorescent microplate assay using specific substrates. The lysosomal number, alkalinity, permeability and cathepsin B activity were determined by flow cytometry with ectodermal dysplasia-1, lysosensor and acridine orange staining, and using cathepsin B specific substrate, respectively.

**Results:**

Confocal imaging revealed that IONPs were markedly engulfed by microglia. Exposure to IONPs attenuated the production of IL-1β, but not TNF-α. Concordantly, the activity of ICE, but not the TACE, was suppressed in IONP-treated cells. Mechanistic studies showed that IONPs accumulated in lysosomes and the number of lysosomes was increased in IONP-treated cells. In addition, exposure to IONPs increased lysosomal permeability and alkalinity, but decreased the activity of cathepsin B, a secretory lysosomal enzyme involved in the activation of ICE.

**Conclusions:**

Our results demonstrated a contrasting effect of IONPs on the production of IL-1β and TNF-α by LPS-stimulated microglia, in which the attenuation of IL-1β by IONPs was mediated by inhibiting the secretory lysosomal pathway of cytokine processing.

## Background

Iron oxide nanoparticles (IONPs) have been employed for a variety of biomedical research and diagnostic purposes, including cancer treatment, cell labeling, drug delivery and magnetic resonance imaging (MRI) [1-4]. Previous studies reported that IONPs affected the viability and functionality of macrophages, including the induction of oxidative stress and apoptosis, and the suppression of phagocytic activity and cytokine production [5-9]. In addition, IONPs have been shown capable of crossing the blood-brain-barrier, rendering IONPs a promising imaging agent for the diagnosis of neuroinflammation and brain injury [10-12]. In light of the increasingly applications of IONPs for the diagnostic imaging of the central nervous system (CNS), the interaction between brain cells and IONPs is a relevant issue to be addressed. To date, it remains mostly elusive if IONPs influence the functionality of central immune cells, such as microglia.

Microglia are the brain-resident macrophage-like immunocompetent cells responsible for the surveillance of homeostasis in the CNS. In response to pathogen infection, foreign agent invasion and injury, microglia are rapidly activated, and undergo morphologic and functional alterations, including proliferation, migration to the site of inflammation, and phagocytosis of foreign agents and cell debris. In addition, activated microglia produce pro-inflammatory cytokines and cytotoxic factors, including interleukin (IL)-1β, nitric oxide (NO), tumor necrosis factor (TNF)-α and reactive oxygen species (ROS) [13,14]. These mediators play a key role in the prevention of brain cells from further damage and to promote the repair of the damaged tissue. For example, IL-1β produced by activated microglia enhances the proliferation of astrocytes, stimulates neovascularization and promotes the repair of nervous tissues [15-18]. Microglial TNF-α can promote neural cell survival and proliferation, and enhance the release of glutamate from astrocytes [19,20]. Previous studies reported that intranasal administration of gold nanoparticles to mice induced microglial activation and internalization of gold nanoparticles, and a transient up-regulation of Toll-like receptor-2 in the olfactory bulb [21]. In addition, intranasal exposure of mice to IONPs resulted in the transportation of the nanoparticles into the brain *via* the olfactory route, and induced the recruitment, activation and proliferation of microglia cells in the brain. Exposure of BV2 microglial cells to IONPs elicited a marked production of ROS and NO. IONPs were also found to be engulfed by BV2 cells, which induced a large number of cellular vesicles, swelling of endoplasmic reticulum and morphological alterations of mitochondrial cristae [22]. Collectively, these results indicate that the functionality and morphology of resting microglia are altered in response to nanoparticle exposure. Microglia play a pivotal role in neuroinflammation, in which they can be activated by various stimuli, such as lipopolysaccharides (LPS) derived from Gram-negative bacteria. To date, evidence pertaining to the potential impact of IONPs on the functionality of activated microglia is scarce. The objective of the present study was to investigate the effect of IONPs on the expression of pro-inflammatory cytokines by LPS-activated microglia. Here, we reported that IONPs suppressed the production of IL-1β by activated microglia *via* the secretory lysosomal pathway of cytokine processing.

## Results and discussion

### Characterization of IONPs and uptake of IONPs by primary microglia

**Figure 1:**
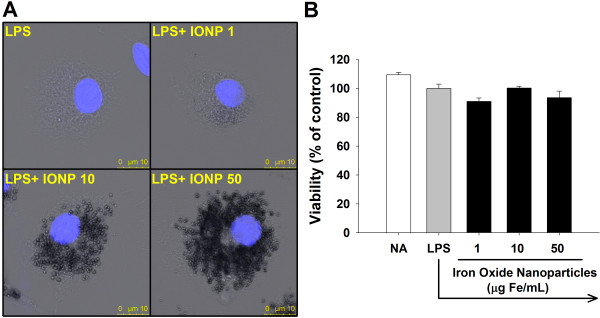
**Exposure to iron oxide nanoparticles (IONPs) did not cause cytotoxicity to primary microglial cells.** Primary microglial cells (4 × 10^5^ cells/mL) were either left untreated (naïve; NA), or pretreated with IONPs (1–50 μg Fe/mL) for 30 min followed by stimulation with LPS (100 ng/mL) for 24 h. **(A)** The nuclei of cells pretreated with IONPs and stimulated with LPS were visualized by confocal microscopy with Hoechst (blue) staining. In the bright field, cells treated with IONPs show numerous dark brown dots accumulated intracellularly. **(B)** The cell viability was determined by the MTT assay. Data are expressed as the mean ± SE of triplicate cultures. Results are a representative of three independent experiments.

The present study employed the commercial preparation of carboxydextran-coated IONPs Resovist® that has been used in clinical as an imaging contrasting agent. The crystalline core of Resovist® is composed of magnetite (Fe_3_O_4_) and maghemite (Fe_2_O_3_). According to the package insert of Resovist®, the hydrodynamic diameters of the nanoparticles range between 45–60 nm. Our confirmatory experiments revealed that Resovist® exhibited a monodisperse population of particles with an average diameter of 58.7 nm in saline [23]. The zeta potentials of the particles in saline and in the culture medium were −13.9 mV and −9.01 mV, respectively. IONPs in culture medium remained a similar net negative-charge as in the serum-free saline. Primary microglial cells were pretreated with IONPs (1–50 μg Fe/mL), and then stimulated with LPS (100 ng/mL) for 24 h. Confocal microscopy was used to monitor the uptake of IONPs, and the images showed the accumulation of dark brown dots in the cytoplasm of cells exposed to IONPs (Figure 1A). These results confirmed the uptake of IONPs by phagocytic cells [9,24-26].

### IONPs did not affect the viability of primary microglia

Although IONPs are generally considered biocompatible, high concentrations of IONPs have been reported to cause cytotoxicity in several glial lines [27]. Moreover, IONPs induced the disappearance of mitochondrial cristae and swelling of endoplasmic reticulum (ER) in BV2 microglial cells [22]. Five-day exposure to IONPs elicited ROS-mediated apoptosis in human macrophages [6]. Other metal nanoparticles such as titanium dioxide also induced apoptosis in murine N9 microglial cells [28]. It is currently unclear whether IONPs affect the viability of primary microglia. Hence, the cell viability was measured using the 3-(4,5-dimethylthiazol-2-yl)-2,5-diphenyl-tetrazolium bromide (MTT) assay. Exposure to IONPs (1–50 μg Fe/mL) for 24 h did not affect cell viability compared to the LPS-stimulated control group (Figure 1B), and the morphology of cells appeared unchanged (Figure 1A). These results are in line with previous results showing that a 24-h exposure of macrophages to carboxydextran-coated IONPs induced no cytotoxicity [6,9,29].

### IONPs differentially modulated the production of proinflammatory cytokines by LPS-stimulated microglia

**Figure 2:**
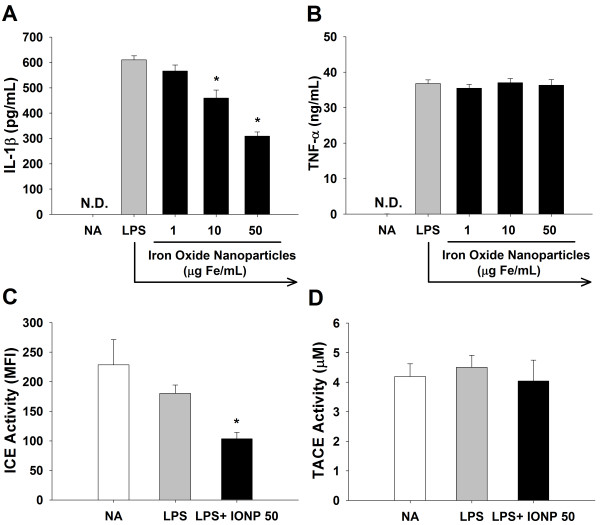
**IONPs differentially affected the production of IL-1β and TNF-α, and the activity of ICE and TACE in LPS-stimulated microglia.** Primary microglial cells (4 × 10^5^ cells/mL) were either left untreated (naïve; NA), or pretreated with IONPs (1–50 μg Fe/mL) for 30 min followed by stimulation with LPS (100 ng/mL) for 24 h. **(A** and **B)** The level of IL-1β and TNF-α in the supernatants was measured by ELISA. **(C** and **D)** The cell lysates were incubated with fluorogenic ICE or TACE substrates for 1 h at 37°C. The fluorescence of cleaved substrates was detected using a fluorescent microplate reader. Data are expressed as the mean ± SE of triplicate cultures. N.D., the level of IL-1β and TNF-α was below the limit of detection. ^*^p < 0.05 compared to the LPS group. Results are a representative of three independent experiments. MFI, mean fluorescence intensity.

To address the potential impact of IONPs on the functionality of activated microglia, we examined the production of IL-1β and TNF-α by LPS-stimulated microglia. Exposure to IONPs significantly attenuated the production of IL-1β, but not TNF-α (Figure 2A and 2B). The maturation of IL-1β and TNF-α requires the proteolytic cleavage of pro-IL-1β and pro-TNF-α by IL-1β converting enzyme (ICE; caspase-1) and TNF-α converting enzyme (TACE), respectively [30,31]. Hence, the activity of these two enzymes was measured using specific fluorescent substrates. Consistent with the differential effect on cytokine production, exposure to IONPs (50 μg/mL) significantly attenuated the activity of ICE, but not TACE (Figure 2C and 2D). These results provide the first evidence to show that direct exposure to IONPs differentially modulated the production of IL-1β and TNF-α by LPS-stimulated microglia.

Cytokines can be processed *via* the classical or nonclassical secretory pathway. In particular, TNF-α is released through the classical pathway, in which newly synthesized pro-TNF-α was trafficked from the ER through the Golgi to recycling endosomes, which fused with cell membrane for the cleavage of pro-TNF-α by TACE and then exocytotic release of mature TNF-α [32,33]. In contrast to the processing of TNF-α, IL-1β lacks the *N*-terminal signal sequence required for ER entry and thus is released through the non-classical pathway independent of ER/Golgi trafficking [34]. Several mechanisms associated with the release of IL-1β have been proposed, including the exocytosis of IL-1β containing secretory lysosomes, shedding of plasma membrane microvesicles, release of IL-1β exosomes from multivesicular bodies and export of IL-1β through ATP-binding cassette transporters [35,36]. Previous studies showed that silica-based nanoparticles inhibited the mRNA expression of TNF-α by microglia [37]. Interestingly, our data revealed that exposure of LPS-stimulated microglia to IONPs inhibited ICE activity and IL-1β secretion, whereas TACE and TNF-α were unaltered (Figure 2), indicating that the non-classical secretory pathway, rather than the ER/Golgi classical secretory pathway, was affected by IONPs.

### IONPs located in lysosomes and increased the number of lysosomes

**Figure 3:**
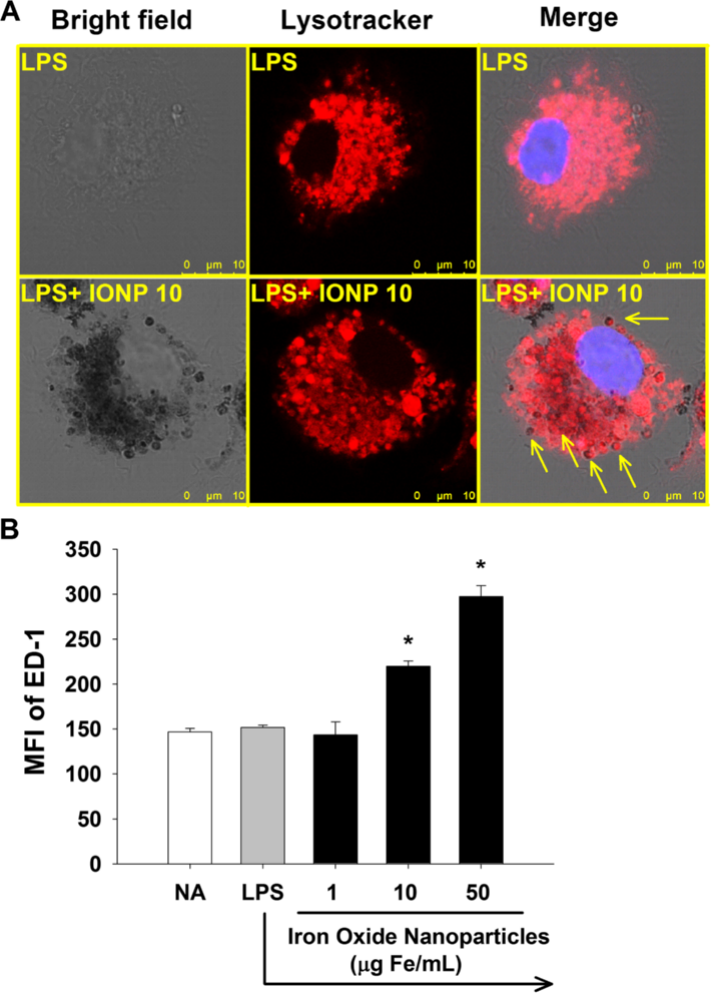
**IONPs located in lysosomes and increased the number of lysosomes in LPS-stimulated microglia.** Primary microglial cells (4 × 10^5^ cells/mL) were either left untreated (naïve; NA), or pretreated with IONPs (1–50 μg Fe/mL) for 30 min followed by stimulation with LPS (100 ng/mL) for 24 h. **(A)** The lysosomes of cells were visualized by confocal microscopy with LysoTracker (red) staining. Cells treated with IONPs show an extensive distribution of the internalized dark brown dots of IONPs. Arrows indicate the colocalization of some dark brown spots and lysosomes. **(B)** The expression of ED-1 was measured by flow cytometry. Data are expressed as the mean ± SE of triplicate cultures. Results are a representative of three independent experiments. ^*^p < 0.05 compared to the LPS group. MFI, mean fluorescence intensity.

Previous studies reported the accumulation of IONPs in lysosomes of macrophages [38]. Gold nanoparticles also accumulated in lysosomes of N9 microglial cells [21], implicating that lysosomes may be a critical intracellular target for nanoparticles in phagocytic cells. In addition, it has been shown that the lysosomal enzyme cathepsin B plays a key role in the processing and maturation of IL-1β in activated microglia [39]. To elucidate the underlying mechanisms for IONP-mediated differential effects on the production of IL-1β and TNF-α, the functionality of lysosomes was studied. The intracellular distribution of IONPs was examined by confocal microscopy using the lysosomal marker LysoTracker™ Red DND-99. We revealed that many of dark brown dots in the cytoplasm were colocalized with the red fluorescence of LysoTracker™, demonstrating the distribution of IONPs in lysosomes (Figure 3A). We also observed that some lysosomes did not contain nanoparticle dots, and that some internalized IONPs did not colocalize with lysosomes (Figure 3A). Hence, not all IONPs were located in lysosomes. Previous results showed that IONPs could be endocytosed by macrophages and microglia [22,38,40,41]. We therefore speculate that IONPs may distribute in cytoplasmic compartments other than lysosomes, such as endosomes, which may account for the observed non-lysosomal distribution of IONPs. Next, we investigated the effect of IONPs on the number of lysosomes by measuring the expression of the macrophage/microglia specific lysosomal membrane protein ectodermal dysplasia (ED)-1. Exposure to IONPs markedly increased the expression of ED-1 (Figure 3B), indicating that the number of lysosomes was increased. Our results are in agreement with previous results showing that intranasal exposure of mice to IONPs resulted in an increased number of lysosomes in microglia [22].

### IONPs attenuated the activity of cathepsin B

**Figure 4:**
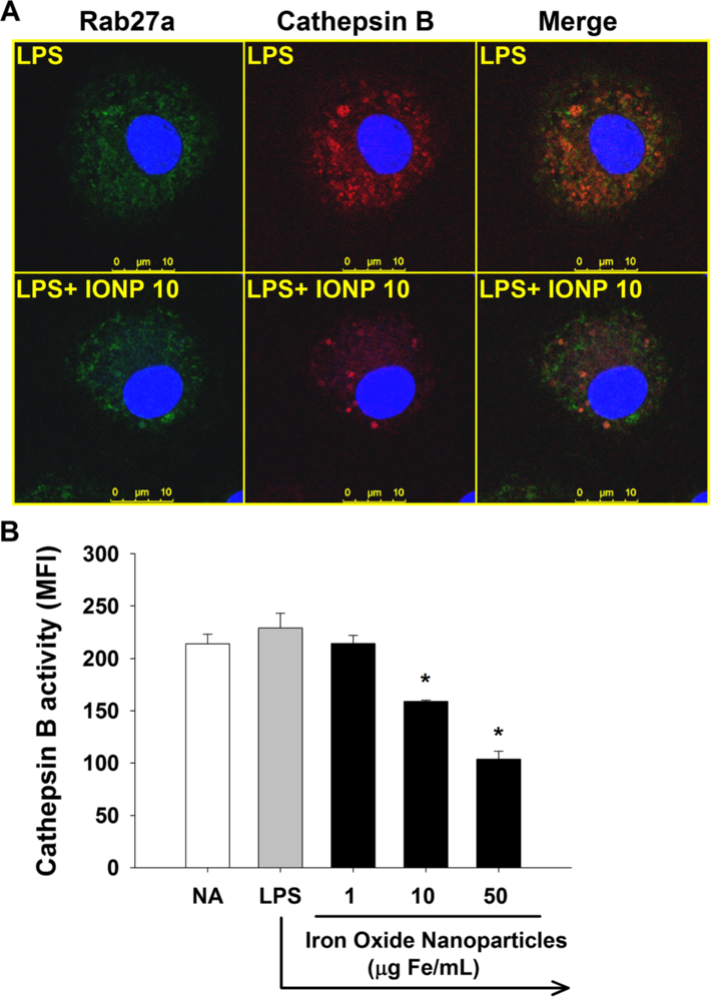
**IONPs attenuated the cathepsin B enzyme activity of secretory lysosomes in LPS-stimulated microglia.** Primary microglial cells (4 × 10^5^ cells/mL) were either left untreated (naïve; NA), or pretreated with IONPs (1–50 μg Fe/mL) for 30 min followed by stimulation with LPS (100 ng/mL) for 24 h. **(A)** The cells pretreated with IONPs and stimulated with LPS were incubated with the cathepsin B substrate (red) for 1 h at 37°C and then stained for the secretory lysosome marker Rab27a (green). The fluorescence was visualized by confocal microscopy. Note the presence of orange signals in the merged images indicating the colocalization of cathepsin B and Rab27a. **(B)** The red fluorescence of 5000 single cells was measured by flow cytometry. Data are expressed as the mean ± SE of triplicate cultures. Results are a representative of three independent experiments. ^*^p < 0.05 compared to the control group. MFI, mean fluorescence intensity.

Secretory lysosomes serve both as a degradative and as a secretory compartment. To date, most cell types containing secretory lysosomes are hematopoietic lineage-derived immunocytes, including dendritic cells, lymphocytes, macrophages, *etc.*[42,43]. It remains unclear if microglia possess secretory lysosomes. The secretory lysosomes were examined by determining the expression of Rab27a, a small GTPase involved in the fusion of secretory lysosomes with plasma membrane during the final stage of cytokine exocytosis [43,44]. In addition to lysosomal exocytosis, Rab27a has been associated with exosome secretion [45]. As shown in Figure 4A, the merge image of LPS-stimulated microglia showed a diffuse distribution of orange signals, demonstrating the colocalization of cathepsin B and Rab27a. Notably, very few red (cathepsin B-single positive) signals were observed in the merged image, suggesting that cathepsin B was primarily located in Rab27a-positive sites. As cathepsin B is a critical lysosomal protease [46,47], these results suggest that the colocalization signals primarily represent secretory lysosomes, rather than exosomes. We also observed some green (Rab27a-single positive) signals in the merged image, demonstrating that some of Rab27a-positive sites were not lysosomes, which might be exosomes. Further studies are required to address this notion. We further observed that exposure to IONPs markedly attenuated red (cathepsin B-single positive) and the merged orange signals (Figure 4A). Quantitative data from flow cytometry confirmed that exposure to IONPs (10–50 μg Fe/mL) significantly attenuated the cathepsin B activity (Figure 4B). These results showed for the first time that IONPs attenuated the cathepsin B activity in the secretory lysosomes of microglia. As cathepsin B is involved in the processing of IL-1β in activated microglia [39], we speculate that IONPs may affect the secretory lysosomal pathway of IL-1β processing.

### IONPs elevated lysosomal alkalinity and permeability

**Figure 5:**
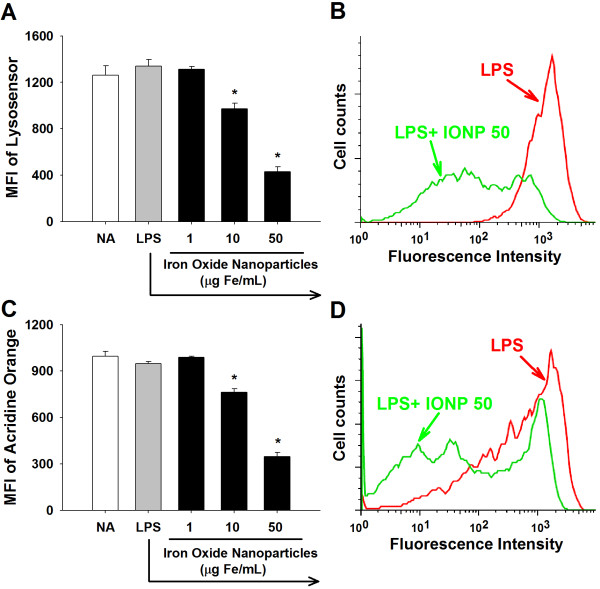
**IONPs increased the alkalinity and permeability of lysosomes in LPS-stimulated microglia.** Primary microglial cells (4 × 10^5^ cells/mL) were either left untreated (naïve; NA), or pretreated with IONPs (1–50 μg Fe/mL) for 30 min followed by stimulation with LPS (100 ng/mL) for 24 h. **(A** and **B)** The acidity of lysosomes was evaluated by flow cytometry with lysosensor Green DND-189 staining. **(C** and **D)** The permeability of microglial cells were measured by flow cytometry with acridine orange staining. (B and D) Representative histograms of lysosensor and acridine orange in cells pretreated with IONPs (50 μg Fe/mL) and stimulated with LPS were shown. Data are expressed as the mean ± SE of triplicate cultures. Results are a representative of three independent experiments. ^*^p < 0.05 compared to the LPS group. MFI, mean fluorescence intensity.

The acid environment in lysosomes is crucial for the optimal function of lysosomal enzymes [48]. The acidity of lysosomes was measured by flow cytometry using LysoSensor Green DND-189, which emits fluorescence in acidic organelles, such as lysosomes [49]. The fluorescent intensity of LysoSensor was markedly attenuated in IONP-treated cells (Figure 5A and 5B), indicating an increased pH in lysosomes. We next examined whether IONPs affected the permeability of lysosomes using acridine orange that can be trapped in lysosomes and emits red fluorescence. Exposure of microglia to IONPs attenuated acridine orange fluorescence (Figure 5C and 5D), indicating an increased lysosomal permeability. Collectively, these results suggest that exposure to IONPs might impair lysosomal functions by increasing the lysosomal permeability and alkalinity in LPS-stimulated microglia. Our findings are in line with previous results showing that gold nanoparticles impair lysosomal degradation by elevating the lysosomal alkalinity in rat normal kidney cells [49]. Moreover, the disruption of lysosomal integrity could be a critical mechanism contributing to IONP-mediated suppression of the secretory lysosomal processing of IL-1β production in LPS-stimulated microglia.

IL-1β is one of the major pro-inflammatory cytokine produced by activated microglia, which induces the production of IL-6 and NO in astrocytes [16,17], and increase oxidative activity in microglia [50]. In addition, IL-1β released by microglia induces the proliferation of astrocytes, stimulates neovascularization and promotes repair of the CNS in brain injury [15,18]. In the present study, we showed that IONPs attenuated IL-1β production by suppressing the secretory lysosomal functionality in LPS-stimulated microglia. These results suggest that IONPs might impair the host defense against pathogens and the repairing capacity in the CNS. In addition, microglia are the major immunocompetent cells possessing antigen-presenting functions in the CNS [16]. It has been shown that lysosomal cysteine proteases, including cathepsin B, are involved in the regulation of antigen processing in APC [51]. Our data showing the suppression of cathepsin B activity by IONPs suggest that the microglial capability of antigen processing may also be affected. Further studies are warranted to address this issue.

## Conclusions

The present study showed that IONPs accumulated in lysosomes and suppressed the production of IL-1β by affecting the secretory lysosomal pathway of cytokine processing in LPS-stimulated microglia. These findings provide new insights to the potential impact of IONP exposure on the functionality of activated microglia and indicate lysosomes as a crucial intracellular target for IONPs. Together with previous reports showing various effects on microglial functionality by other nanoparticles, including gold, silica, and titanium oxide [21,37,49,52], exposure to nanoparticles may cause microglial dysfunction leading to deleterious influence on the central immune homeostasis.

## Methods

### Materials

All reagents were purchased from Sigma Chemical (St Louis, MO) unless otherwise stated. Resovist®, a commercial preparation of carboxydextran-coated iron oxide nanoparticles (IONPs) containing 28 mg Fe/mL, was obtained from Schering AG (Berlin, Germany). Dulbecco’s Modified Eagle Medium (DMEM) was obtained from Caisson Laboratories (Rexburg, ID). Fetal bovine serum (FBS), horse serum (HS) and cell culture reagents were purchased from GIBCO BRL (Gaithersburg, MD). Enzyme-linked immunosorbent assay (ELISA) sets for cytokine measurement were purchased from BD Biosciences (San Diego, CA). ICE/Caspase 1 protease assay kit and fluorogenic substrates Ac-YVAD-AFC (7-amino-4-methylcoumarin) were obtained from Chemicon (Temecula, CA) and Tocris (Bristol, UK), respectively. TACE fluorogenic activity assay kit was purchased from AnaSpec (San Jose, CA). Magic Red™ cathepsin B detection kit was purchased from Immunochemistry Technologies (Bloomington, MN).

### Culture of primary murine microglial cells

Primary mix glial cultures were prepared as previously described with minor modifications [53]. Briefly, after decapitation, forebrains of new-born BALB/c mice were dissociated mechanically. Their meninges were removed aseptically and the brain cells were resuspended in DMEM containing 10% FBS, 10% HS, 4 mM _L_-glutamine, 100 U/mL penicillin and 100 μg/mL streptomycin. The cells were cultured on poly-_D_-lysine (25 μg/mL)-coated flasks, and medium was replenished 6–8 days after initial seeding. Upon reaching confluence (12−16 days), loosely adherent microglial cells were collected by shaking at 165 rpm at 37°C for 3 h. The enriched microglial cells were cultured on culture plates for 2 h to allow attachment, and then the medium was changed to DMEM containing 1% HS, 4 mM _L_-glutamine, 100 U/mL penicillin and 100 μg/mL streptomycin for experiments with IONPs exposure. In all cases, the cells were cultured at 37°C in 5% CO_2_. The purity of microglial culture was > 90% as determined by flow cytometry with CD11b staining.

### Characterization of IONPs

The particle size distribution and zeta potential of the IONPs were measured by phase analysis light scattering using Zetasizer nano-ZS (Malvern Instruments Ltd, Malvern, Worcestershire, UK). IONPs were appropriately diluted with 0.22 μm-filtered saline for size measurement at 25°C. The zeta potentials of IONPs in saline and culture medium (DMEM containing 1% horse serum) were measured at 25°C.

### Confocal imaging of engulfed nanoparticles and lysosomes

Microglial cells (4 × 10^5^ cells/mL) cultured on cover slips were pretreated with IONPs (1–50 μg Fe/mL) for 30 min followed by stimulation with LPS (100 ng/mL; *Escherichia coli* O55:B5) for 24 h. The cells were stained with 1 μM LysoTracker™ Red DND-99 (Invitrogen, Carlsbad, CA) for 2 h at 37°C. After washing, the cells were fixed with 4% paraformaldehyde for 15 min at room temperature. For detection of nuclei, the fixed cells were stained with Hoechst 33258 (5 μg/mL) at room temperature for 5 min. After washing, the cells were mounted in ProLong Gold antifade reagent (Invitrogen, Carlsbad, CA) and visualized on a Leica TCS SP5 II scanning confocal microscope (Leica Microsystems, Wetzlar, Germany).

### Measurement of cell viability

The viability of microglia was determined by the 3-(4,5-dimethylthiazol-2-yl)-2,5-diphenyl-tetrazolium bromide (MTT) assay. Microglial cells (4 × 10^5^ cells/mL) were cultured in quadruplicate in a 96-well plate (0.1 mL/well). The cells were either left untreated (naïve; NA), or pretreated with IONPs (1–50 μg Fe/mL) for 30 min followed by stimulation with LPS (100 ng/mL) for 24 h. An MTT stock solution (5 mg/mL) was added to each well (10 μL/well) 4 h before the end of incubation. After washing, the formed formazan was dissolved by the addition of lysis buffer (10% SDS in *N,N*-dimethylformamide; 200 μL/well). The absorbance of the formazan product was measured at 570 nm and at 630 nm as a background reference, using a microplate reader (Dynatech Laboratories, Chantilly, VA).

### Measurement of cytokines

Microglial cells (4 × 10^5^ cells/mL) were cultured in tripricate in a 48-well plate (0.25 mL/well) and treated with IONPs and LPS as described above in the MTT assay. The supernatants were collected and quantified for IL-1β and TNF-α by standard sandwich ELISA.

### Measurement of ICE and TACE activity

Microglial cells (4 × 10^5^ cells/mL) were cultured in a 6-cm dish (5.5 mL/dish) and treated with IONPs and LPS as described above in the MTT assay. After washing, the cells were harvested and lysed with cell lysis buffer. The lysates were incubated with 200 μM of the ICE fluorogenic substrates Ac-YVAD-AFC and 5 mM of DTT for 1 h at 37°C, and the fluorescence was measured at 400 nm excitation and 505 nm emissions. The cell lysates were also incubated with 40 μM of the TACE fluorogenic substrate for 1 h at 37°C in the dark with gentle shaking, and the fluorescence was detected at 490 nm excitation and 520 nm emissions.

### Analysis of the amount of lysosomes

Microglial cells (4 × 10^5^ cells/mL) were cultured in tripricate in a 24-well plate (0.5 mL/well) and treated with IONPs and LPS as described above in the MTT assay. The cells were fixed in 4% paraformaldehyde, permeabilized with 0.2% Trion-X and incubated in blocking buffer containing 2.5% BSA in PBS for 30 min at 4°C. The cells were then stained with appropriately diluted APC-labeled rat anti-mouse ED-1 antibody (BioLegend, CA) for 1 h at 4°C in the dark. After washing, the fluorescence of 5000 single cells for each sample was measured using a flow cytometer at emission of 670 nm (BD LSRFortessa, San Jose, CA). The data were analyzed using the software Flowjo 5.7.

### Measurement of lysosomal acidity and permeability

The acidity and permeability of lysosome were determined by flow cytometry using LysoSensor™ Green DND-189 and acridine orange, respectively. Microglial cells (4 × 10^5^ cells/mL) were cultured in tripricate in a 48-well plate (0.25 mL/well) and treated with IONPs and LPS as described above in the MTT saasy. The cells were stained with 1 μM Lysosensor™ for 2 h or 5 μg/mL acridine orange for 10 min at 37°C. After washing, the fluorescence of 5000 single cells for each sample was measured using a flow cytometer at emission of 525 nm and 610 nm for Lysosensor™ and acridine orange, respectively.

### Detection of cathepsin B enzyme activity and secretory lysosomes

Microglial cells (4 × 10^5^ cells/mL) were cultured in tripricate in a 48-well plate (0.25 mL/well) and treated with IONPs and LPS as described above in the MTT assay. The cells were stained with Magic Red™ cathepsin B substrate for 1 h at 37°C. Once the substrate is cleaved by active cathepsin B, its product emits red fluorescence whose intensity is a direct measurement of the enzymatic activity of cathepsin B. After washing, the fluorescence of 5000 single cells for each sample was measured using a flow cytometer at emission of 610 nm and visualized on a Leica TCS SP5 II scanning confocal microscope. The secretory lysosomes in microglia were stained with appropriately diluted Alexa Fluor 488-labeled rabbit anti-Rab27a antibody (Bioss, MA) and visualized by confocal microscopy as described above for ED-1 staining.

### Statistical analysis

The mean ± standard error (SE) was determined for each treatment group in the individual experiments. Homogeneous data were evaluated by a parametric analysis of variance, and Dunnett’s two-tailed *t-*test was used to compare treatment groups to the control group. *P* value < 0.05 was defined as statistical significance.

## Abbreviations

APC: Antigen-presenting cells; CNS: Central nervous system; DMEM: Dulbecco’s Modified Eagle Medium; ED-1: Ectodermal dysplasia-1; ELISA: Enzyme-linked immunosorbent assay; ER: Endoplasmic reticulum; FBS: Fetal bovine serum; HS: Horse serum; ICE: IL-1β converting enzyme; IL: Interleukin; IONPs: Iron oxide nanoparticles; LPS: Lipopolysaccharide; MFI: Mean fluorescence intensity; MRI: Magnetic resonance imaging; MTT: 3-(4,5-dimethylthiazol-2-yl)-2,5-diphenyl-tetrazolium bromide; NO: Nitric oxide; ROS: Reactive oxygen species; TACE: TNF-α converting enzyme; TNF: Tumor necrosis factor.

## Competing interests

The authors declare that they have no competing interests.

## Authors’ contributions

HYW and MCC designed the research, performed experiments and drafted the paper. CCW and CHH provided technical aid for confocal imaging and advices for statistical analysis. HJL and TRJ provided advices for designing the research and interpreting the results, and contributed to write the manuscript. All authors read and approved the final manuscript.

## References

[B1] Xie J Huang J Li X Sun S Chen X Iron oxide nanoparticle platform for biomedical applications Curr Med Chem 2009 16 1278 1294 10.2174/092986709787846604 19355885

[B2] Yu MK Jeong YY Park J Park S Kim JW Min JJ Kim K Jon S Drug-loaded superparamagnetic iron oxide nanoparticles for combined cancer imaging and therapy in vivo Angew Chem Int Ed Engl 2008 47 5362 5365 10.1002/anie.200800857 18551493

[B3] Liong M Lu J Kovochich M Xia T Ruehm SG Nel AE Tamanoi F Zink JI Multifunctional inorganic nanoparticles for imaging, targeting, and drug delivery ACS Nano 2008 2 889 896 10.1021/nn800072t 19206485 PMC2751731

[B4] Chouly C Pouliquen D Lucet I Jeune JJ Jallet P Development of superparamagnetic nanoparticles for MRI: effect of particle size, charge and surface nature on biodistribution J Microencapsul 1996 13 245 255 10.3109/02652049609026013 8860681

[B5] Shen CC Wang CC Liao MH Jan TR A single exposure to iron oxide nanoparticles attenuates antigen-specific antibody production and T-cell reactivity in ovalbumin-sensitized BALB/c mice Int J Nanomedicine 2011 6 1229 1235 21753874 10.2147/IJN.S21019 PMC3131189

[B6] Lunov O Syrovets T Buchele B Jiang X Rocker C Tron K Nienhaus GU Walther P Mailander V Landfester K Simmet T The effect of carboxydextran-coated superparamagnetic iron oxide nanoparticles on c-Jun N-terminal kinase-mediated apoptosis in human macrophages Biomaterials 2010 31 5063 5071 10.1016/j.biomaterials.2010.03.023 20381862

[B7] Chen BA Jin N Wang J Ding J Gao C Cheng J Xia G Gao F Zhou Y Chen Y The effect of magnetic nanoparticles of Fe(3)O(4) on immune function in normal ICR mice Int J Nanomedicine 2010 5 593 599 20856834 10.2147/ijn.s12162 PMC2939704

[B8] Cho WS Cho M Kim SR Choi M Lee JY Han BS Park SN Yu MK Jon S Jeong J Pulmonary toxicity and kinetic study of Cy5.5-conjugated superparamagnetic iron oxide nanoparticles by optical imaging Toxicol Appl Pharmacol 2009 239 106 115 10.1016/j.taap.2009.05.026 19520096

[B9] Hsiao JK Chu HH Wang YH Lai CW Chou PT Hsieh ST Wang JL Liu HM Macrophage physiological function after superparamagnetic iron oxide labeling NMR Biomed 2008 21 820 829 10.1002/nbm.1260 18470957

[B10] Weinstein JS Varallyay CG Dosa E Gahramanov S Hamilton B Rooney WD Muldoon LL Neuwelt EA Superparamagnetic iron oxide nanoparticles: diagnostic magnetic resonance imaging and potential therapeutic applications in neurooncology and central nervous system inflammatory pathologies, a review J Cereb Blood Flow Metab 2010 30 15 35 10.1038/jcbfm.2009.192 19756021 PMC2949106

[B11] Thorek DL Weisshaar CL Czupryna JC Winkelstein BA Tsourkas A Superparamagnetic iron oxide-enhanced magnetic resonance imaging of neuroinflammation in a rat model of radicular pain Mol Imaging 2011 10 206 214 21496449

[B12] Jin AY Tuor UI Rushforth D Filfil R Kaur J Ni F Tomanek B Barber PA Magnetic resonance molecular imaging of post-stroke neuroinflammation with a P-selectin targeted iron oxide nanoparticle Contrast Media Mol Imaging 2009 4 305 311 10.1002/cmmi.292 19941323

[B13] Aloisi F Immune function of microglia Glia 2001 36 165 179 10.1002/glia.1106 11596125

[B14] Kreutzberg GW Microglia: a sensor for pathological events in the CNS Trends Neurosci 1996 19 312 318 10.1016/0166-2236(96)10049-7 8843599

[B15] Giulian D Lachman LB Interleukin-1 stimulation of astroglial proliferation after brain injury Science 1985 228 497 499 10.1126/science.3872478 3872478

[B16] Gehrmann J Matsumoto Y Kreutzberg GW Microglia: intrinsic immuneffector cell of the brain Brain Res Brain Res Rev 1995 20 269 287 10.1016/0165-0173(94)00015-H 7550361

[B17] Lee SC Dickson DW Brosnan CF Interleukin-1, nitric oxide and reactive astrocytes Brain Behav Immun 1995 9 345 354 10.1006/brbi.1995.1032 8903851

[B18] Mason JL Suzuki K Chaplin DD Matsushima GK Interleukin-1beta promotes repair of the CNS J Neurosci 2001 21 7046 7052 11549714 10.1523/JNEUROSCI.21-18-07046.2001 PMC6762979

[B19] Smith JA Das A Ray SK Banik NL Role of pro-inflammatory cytokines released from microglia in neurodegenerative diseases Brain Res Bull 2012 87 10 20 10.1016/j.brainresbull.2011.10.004 22024597 PMC9827422

[B20] Hanisch UK Microglia as a source and target of cytokines Glia 2002 40 140 155 10.1002/glia.10161 12379902

[B21] Hutter E Boridy S Labrecque S Lalancette-Hebert M Kriz J Winnik FM Maysinger D Microglial response to gold nanoparticles ACS Nano 2010 4 2595 2606 10.1021/nn901869f 20329742

[B22] Wang Y Wang B Zhu MT Li M Wang HJ Wang M Ouyang H Chai ZF Feng WY Zhao YL Microglial activation, recruitment and phagocytosis as linked phenomena in ferric oxide nanoparticle exposure Toxicol Lett 2011 205 26 37 10.1016/j.toxlet.2011.05.001 21596115

[B23] Shen CC Liang HJ Wang CC Liao MH TRJ A role of cellular glutathione in the differential effects of iron oxide nanoparticles on antigen-specific T cell cytokine expression Int J Nanomedicine 2011 6 2791 2798 22114506 10.2147/IJN.S25588 PMC3218589

[B24] Mou Y Chen B Zhang Y Hou Y Xie H Xia G Tang M Huang X Ni Y Hu Q Influence of synthetic superparamagnetic iron oxide on dendritic cells Int J Nanomedicine 2011 6 1779 1786 21980240 10.2147/IJN.S23240 PMC3184937

[B25] Yang CY Tai MF Lin CP Lu CW Wang JL Hsiao JK Liu HM Mechanism of cellular uptake and impact of ferucarbotran on macrophage physiology PloS one 2011 6 e25524 10.1371/journal.pone.0025524 21991395 PMC3182225

[B26] Yeh CH Hsiao JK Wang JL Sheu F Immunological impact of magnetic nanoparticles (Ferucarbotran) on murine peritoneal macrophages J Nanoparticle Res : Interdiscip Forum Nanoscale Sci Technol 2010 12 151 160

[B27] Ankamwar B Lai TC Huang JH Liu RS Hsiao M Chen CH Hwu YK Biocompatibility of Fe(3)O(4) nanoparticles evaluated by in vitro cytotoxicity assays using normal, glia and breast cancer cells Nanotechnol 2010 21 75102 10.1088/0957-4484/21/7/075102 20090199

[B28] Li X Xu S Zhang Z Schluesener H Apoptosis induced by titanium dioxide nanoparticles in cultured murine microglia N9 cells Chin Sci Bull 2009 54 3830 3836 10.1007/s11434-009-0548-x

[B29] Yeh C-H Hsiao J-K Wang J-L Sheu F Immunological impact of magnetic nanoparticles (Ferucarbotran) on murine peritoneal macrophages J Nanopart Res 2010 12 151 160 10.1007/s11051-009-9589-y

[B30] Chauvet N Palin K Verrier D Poole S Dantzer R Lestage J Rat microglial cells secrete predominantly the precursor of interleukin-1beta in response to lipopolysaccharide Eur J Neurosci 2001 14 609 617 10.1046/j.0953-816x.2001.01686.x 11556886

[B31] Black RA Tumor necrosis factor-alpha converting enzyme Int J Biochem Cell Biol 2002 34 1 5 10.1016/S1357-2725(01)00097-8 11733179

[B32] Stow JL Low PC Offenhauser C Sangermani D Cytokine secretion in macrophages and other cells: pathways and mediators Immunobiol 2009 214 601 612 10.1016/j.imbio.2008.11.005 19268389

[B33] Lacy P Stow JL Cytokine release from innate immune cells: association with diverse membrane trafficking pathways Blood 2011 118 9 18 10.1182/blood-2010-08-265892 21562044

[B34] Nickel W The mystery of nonclassical protein secretion. A current view on cargo proteins and potential export routes Eur J Biochem 2003 270 2109 2119 10.1046/j.1432-1033.2003.03577.x 12752430

[B35] Nickel W Rabouille C Mechanisms of regulated unconventional protein secretion Nat Rev Mol Cell Biol 2009 10 148 155 10.1038/nrm2617 19122676

[B36] Eder C Mechanisms of interleukin-1beta release Immunobiol 2009 214 543 553 10.1016/j.imbio.2008.11.007 19250700

[B37] Choi J Zheng Q Katz HE Guilarte TR Silica-based nanoparticle uptake and cellular response by primary microglia Environ Health Perspect 2010 118 589 595 20439179 10.1289/ehp.0901534 PMC2866671

[B38] Lunov O Syrovets T Rocker C Tron K Nienhaus GU Rasche V Mailander V Landfester K Simmet T Lysosomal degradation of the carboxydextran shell of coated superparamagnetic iron oxide nanoparticles and the fate of professional phagocytes Biomaterials 2010 31 9015 9022 10.1016/j.biomaterials.2010.08.003 20739059

[B39] Terada K Yamada J Hayashi Y Wu Z Uchiyama Y Peters C Nakanishi H Involvement of cathepsin B in the processing and secretion of interleukin-1beta in chromogranin A-stimulated microglia Glia 2010 58 114 124 10.1002/glia.20906 19544382

[B40] Lunov O Zablotskii V Syrovets T Rocker C Tron K Nienhaus GU Simmet T Modeling receptor-mediated endocytosis of polymer-functionalized iron oxide nanoparticles by human macrophages Biomaterials 2011 32 547 555 10.1016/j.biomaterials.2010.08.111 20880574

[B41] Luther EM Petters C Bulcke F Kaltz A Thiel K Bickmeyer U Dringen R Endocytotic uptake of iron oxide nanoparticles by cultured brain microglial cells Acta biomaterialia 2013 9 8454 8465 10.1016/j.actbio.2013.05.022 23727247

[B42] Blott EJ Griffiths GM Secretory lysosomes Nat Rev Mol Cell Biol 2002 3 122 131 10.1038/nrm732 11836514

[B43] Griffiths G What’s special about secretory lysosomes? Semin Cell Dev Biol 2002 13 279 284 10.1016/S1084-9521(02)00057-5 12243727

[B44] Elstak ED Neeft M Nehme NT Voortman J Cheung M Goodarzifard M Gerritsen HC van Bergen En Henegouwen PM Callebaut I de Saint Basile G van der Sluijs P The munc13-4-rab27 complex is specifically required for tethering secretory lysosomes at the plasma membrane Blood 2011 118 1570 1578 10.1182/blood-2011-02-339523 21693760

[B45] Ostrowski M Carmo NB Krumeich S Fanget I Raposo G Savina A Moita CF Schauer K Hume AN Freitas RP Rab27a and Rab27b control different steps of the exosome secretion pathway Nat Cell Biol 2010 12 19 30 sup pp 11–13 10.1038/ncb2000 19966785

[B46] Guha S Padh H Cathepsins: fundamental effectors of endolysosomal proteolysis Indian J Biochem Biophys 2008 45 75 90 21086720

[B47] Mort JS Buttle DJ Cathepsin B Int J Biochem Cell Biol 1997 29 715 720 10.1016/S1357-2725(96)00152-5 9251238

[B48] Trombetta ES Ebersold M Garrett W Pypaert M Mellman I Activation of lysosomal function during dendritic cell maturation Science 2003 299 1400 1403 10.1126/science.1080106 12610307

[B49] Ma X Wu Y Jin S Tian Y Zhang X Zhao Y Yu L Liang XJ Gold nanoparticles induce autophagosome accumulation through size-dependent nanoparticle uptake and lysosome impairment ACS Nano 2011 5 8629 8639 10.1021/nn202155y 21974862

[B50] Smith ME van der Maesen K Somera FP Macrophage and microglial responses to cytokines in vitro: phagocytic activity, proteolytic enzyme release, and free radical production J Neurosci Res 1998 54 68 78 10.1002/(SICI)1097-4547(19981001)54:1<68::AID-JNR8>3.0.CO;2-F 9778151

[B51] Honey K Rudensky AY Lysosomal cysteine proteases regulate antigen presentation Nat Rev Immunol 2003 3 472 482 10.1038/nri1110 12776207

[B52] Long TC Tajuba J Sama P Saleh N Swartz C Parker J Hester S Lowry GV Veronesi B Nanosize titanium dioxide stimulates reactive oxygen species in brain microglia and damages neurons in vitro Environ Health Perspect 2007 115 1631 1637 10.1289/ehp.10216 18007996 PMC2072833

[B53] Correa F Docagne F Mestre L Clemente D Hernangomez M Loria F Guaza C A role for CB2 receptors in anandamide signalling pathways involved in the regulation of IL-12 and IL-23 in microglial cells Biochem Pharmacol 2009 77 86 100 10.1016/j.bcp.2008.09.014 18848818

